# Characterisation of osteogenic and vascular responses of hMSCs to Ti-Co doped phosphate glass microspheres using a microfluidic perfusion platform

**DOI:** 10.1177/2041731420954712

**Published:** 2020-10-24

**Authors:** Carlotta Peticone, David De Silva Thompson, Nikolay Dimov, Ben Jevans, Nick Glass, Martina Micheletti, Jonathan C Knowles, Hae-Won Kim, Justin J Cooper-White, Ivan B Wall

**Affiliations:** 1 Department of Biochemical Engineering, University College London, London, UK; 2 Centre for Engineering Research, University of Hertfordshire, Hatfield, Hertfordshire, UK; 3 Great Ormond Street Institute of Child Health, University College London, London, UK; 4 Australian Institute for Bioengineering and Nanotechnology, University of Queensland, St. Lucia, Brisbane, Australia; 5 Division of Biomaterials and Tissue Engineering, University College London Eastman Dental Institute, London, UK; 6 The Discoveries Centre for Regenerative and Precision Medicine, UCL Campus, London, UK; 7 Department of Nanobiomedical Science & BK21 PLUS NBM Global Research Center for Regenerative Medicine, Dankook University, Cheonan, Republic of Korea; 8 UCL Eastman-Korea Dental Medicine Innovation Centre, Dankook University, Cheonan, Republic of Korea; 9 Institute for Tissue Regeneration Engineering, Dankook University, Cheonan, Republic of Korea; 10 School of Chemical Engineering, University of Queensland, St. Lucia, Brisbane, Australia; 11 Aston Medical Research Institute and School of Life and Health Sciences, Aston University, Birmingham, UK

**Keywords:** Stem cells, tissue engineering, microfluidics, phosphate glass, osteogenic differentiation

## Abstract

Using microspherical scaffolds as building blocks to repair bone defects of specific size and shape has been proposed as a tissue engineering strategy. Here, phosphate glass (PG) microcarriers doped with 5 mol % TiO_2_ and either 0 mol % CoO (CoO 0%) or 2 mol % CoO (CoO 2%) were investigated for their ability to support osteogenic and vascular responses of human mesenchymal stem cells (hMSCs). Together with standard culture techniques, cell-material interactions were studied using a novel perfusion microfluidic bioreactor that enabled cell culture on microspheres, along with automated processing and screening of culture variables. While titanium doping was found to support hMSCs expansion and differentiation, as well as endothelial cell-derived vessel formation, additional doping with cobalt did not improve the functionality of the microspheres. Furthermore, the microfluidic bioreactor enabled screening of culture parameters for cell culture on microspheres that could be potentially translated to a scaled-up system for tissue-engineered bone manufacturing.

## Introduction

Microspherical scaffolds have been proposed as a potential modular unit for bone tissue engineering applications,^1–5^ as their sphericity could facilitate filling of irregularly shaped defects.^6^ Microspheres have been used both as a tool to expand adherent cells *ex vivo* and also to directly deliver cells to the defect site.^7,8^ Furthermore, being suitable for expansion within a bioreactor system, they are particularly attractive from a biomanufacturing perspective, as they could be scaled up by increasing culture volume and quantities of microspheres within a controlled environment.

Whilst expansion for cell therapy manufacture typically uses inert plastic microcarriers, microspheres for tissue engineering applications need to be made of a suitable implantable biomaterial that supports cell growth but ideally also directs differentiation towards a tissue-specific cell fate and promotes biological responses in vivo.^9^ Several materials including bioactive ceramics, polymers and their composites have been synthesised in the form of microspheres and they have been found to support cell attachment to the curved surface, together with growth and differentiation towards an osteogenic phenotype under specific culture conditions.^9^

The capacity of phosphate glasses to completely dissolve in aqueous solution into non-toxic products, makes them a very attractive material for biomedical applications.^10^ Metal oxides can be used as dopants to control phosphate glass degradation rate, and include compounds such as TiO_2_, Al_2_O_3_, B_2_O_3_, ZnO, MgO, SrO, CuO and Fe_2_O_3_.^11^

In this study, the effect of doping the PG microspheres with titanium and cobalt was investigated, as these ions have been shown to induce osteogenesis^4,12–14^ and angiogenesis,^15–17^ respectively. Ti-doped PG has already been shown to upregulate osteogenic markers in osteosarcoma cells MG63,^18^ as well as human mesenchymal stem cells.^19^ In an in vivo rabbit femoral model, two phosphate glass particles with or without 5% TiO_2_, were mixed with blood and implanted in the defect as a putty.^20^ The macroporous structure of the putty enabled newly formed bone to grow between the particles as the glass was resorbed and to support vessel formation. In a recent study, an ovine model was used to examine the effect of TiO_2_-doping of porous PG microspheres used to treat in bone defects.^21^ While PG lacking Ti induced lower trabeculae-like interconnections and higher fatty bone marrow content, the Ti-doped microspheres were found to have a slower degradation rate and promoted the formation of dense interconnected tissue.

From an angiogenesis perspective, cobalt has been found to have a stabilising effect on HIF1α, inhibiting degradation that usually occurs under normoxic conditions.^22^ This characteristic has been exploited in the tissue engineering field to induce angiogenesis during skeletal regeneration.^23^ Typical responses observed when doping scaffolds made of ceramic materials with different cobalt contents (2%–5% mol) are upregulation of HIF1α and VEGF expression.^16,17,24^ Furthermore, functional responses such as enhanced vessel formation have also been shown in vitro^24,25^ and in vivo.^26,27^

More controversial is the role of Co^2+^ ions on osteogenesis. Osathanon tested the effect of hypoxia on human periodontal ligament cells using cobalt chloride and showed downregulation of osteogenic genes, as well as a significant reduction in mineralisation.^28^ Interestingly, stem cell markers like Oct4 and Rex1 were upregulated in the presence of cobalt, suggesting that cobalt is capable of maintaining a stem-cell state. Similarly, Birgani and colleagues showed reduced ALP activity, BSP gene expression and mineralisation by hMSCs exposed to Co^2+^ ions either dissolved in the cell medium or incorporated to CaP coatings.^29^ On the other hand, Ignjatović et al. reported enhanced bone matrix formation and deposition of calcium, magnesium and phosphorus in a rat osteoporotic defect model that was reconstructed using hydroxyapatite nanoparticles supplemented with the highest content of CoO.^30^

In order to clarify the role of cobalt doping in the context of osteogenesis and angiogenesis, in this study two PG compositions containing 5% mol TiO_2_ either supplemented with 2% mol CoO (CoO 2%) or without (CoO 0%) were studied. The molar concentration of titanium was chosen based on previous studies, showing cytocompatibility and osteogenic potential of 5% mol TiO_2_ doped phosphate glass microspheres.^4,31^ As cobalt-induced cytotoxicity has been reported^32–34^ only one molar concentration previously tested in cobalt-releasing bioactive glass scaffolds was selected, that proved to be safe from a cytotoxic perspective^35^ and to promote in vitro angiogenesis.^15,16^ The effects of soluble ionic species on hMSC and endothelial cells responses were also assessed independently of the physical cell-material interaction. Furthermore, an ex vivo chick embryo chorioallantoic membrane (CAM) assay was also performed to test the capacity of PG microspheres to induce vessel ingrowth, as this assay has been widely used as a platform to screen biomaterial for tissue engineering applications.^36^

In the experiments here described, characterisation of cell-microsphere interactions required transfer of cell-populated microcarriers between multiple dishes, where subsequent cell and tissue level analysis was performed. The processing methods required to do this can lead to disruption of architecture and alter what essential parameters are being measured. Furthermore, growing cells on microspheres in wells only allows to perform static culture.

Within this paper we report how a novel microfluidic device was designed and manufactured with the purpose of assessing how hMSCs and endothelial cells interact with PG microspheres under controlled perfusion conditions and in the absence of manual operations that can be a source of variability and uncertainty in manufacturing. Furthermore, this reduces significantly the amount of material required for testing whilst allowing to screen for a wider spectrum of parameters. Experiments were performed to optimise culturing conditions within the bioreactor, such as the perfusion flow rate. Furthermore, a preliminary screening of microspheres and media composition that promote mesenchymal stem cell proliferation, expression of osteogenic markers and support vascular cells attachment was performed.

## Materials and methods

### Glass preparation and microspheres fabrication

Phosphate glass were prepared by the melt-quench technique from the precursors sodium dihydrogen orthophosphate (NaH_2_PO_4_), calcium carbonate (CaCO_3_), phosphorus pentoxide (P_2_O_5_), titanium dioxide (TiO_2_) and cobalt oxide (CoO) as summarised in Table 1. The glasses were first broken into fragments and then ball-milled and then then sieved down to 63 to 106 mm (Endecotts Ltd.) on a Fritsch Spartan sieve shaker (Fritsch GmbH). The microparticles were spheroidised by passing them through a flame spheroidisation apparatus, as previously described.^4^ Average particle size was found to be 85 µm (data not reported). Material characterisation as well as degradation and ions release studies for the microspheres have been previously published by our group.^35^

**Table 1.** Summary of glass compositions, showing molar concentration (%) of each precursor.

Glass composition (mol %)					
Glass Code	P_2_O_5_	CaO	Na_2_O	TiO_2_	CoO
CoO 0%	45	30	20	5	0
CoO 2%	45	28	20	5	2

CaO: calcium oxide; CoO: cobalt oxide, Na_2_O: sodium oxide, P_2_O_5_: phosphorous pentoxide; TiO_2_: titanium oxide.

### Cell culture

Human bone marrow derived MSCs from multiple donors (Lonza) were obtained from supplier at P2. Cells were seeded at a density of 4000 cells/cm^2^ according to manufacturer instructions in T-175 tissue culture flask in standard growth media (DMEM low glucose (1 g/L) supplemented with 1% Glutamax™ (Gibco™, Life Technologies), 10% FBS (Gibco™, Life Technologies) and 1% antibiotic/antimycotic (A/A) (Gibco™, Life Technologies). Cell cultures were maintained at 37°C/5% CO_2_ and passaged upon reaching confluency using trypsin-EDTA (Gibco™, Life Technologies) solution for 5 min at 37°C. Number of donors used in each assay is reported individually. All experiments were performed with cells at passage P4-P5.

Human umbilical vein endothelial cells (HUVECs) (kind donation from Dr Enca Martin-Rendon’s lab at the University of Oxford) were maintained in EGM™-2 (Lonza) consisting of EBM™-2 supplemented with Bulletkit™ (Lonza) at 37°C and 5% CO_2_. HUVECs were used within passage P4-P6.

#### 2D cell culture (conditioned media)

hMSCs were seeded on 96 well plate tissue culture plastic at a seeding density of 4000 cells/cm^2^ (cell density for expansion) for the negative control group and of 10,000 cells/cm^2^ (cell density for differentiation) for the conditioned media and positive control groups. MSCs were allowed to attach for 24 h in standard growth media before media was changed for each experimental condition as follow:

1. the negative control group was cultured in standard growth media (DMEM + 1% Glutamax + 10% FBS + 1% A/A).
2. the positive control group was cultured in osteoblast differentiation media prepared by supplementing standard growth media with 50 µM ascorbic acid-2 phosphate (Sigma), 10 mM β-glycerolphosphate (Sigma) and 100 nM dexamethasone (Sigma).
3. the microspheres conditioned media group was cultured in media prepared as follow: 30 mg of CoO 0% and CoO 2% PG microspheres were weighed and placed in 24 well plates. Following 1 h 30 min UV sterilisation the microspheres were soaked in 900 µL of standard growth media and incubated at 37°C/5% CO_2_. After 24 h, the conditioned media were aspirated and 0.22 µm filtered.

Media were replaced every 3 days for each condition.

#### 3D cell culture (microspheres)

For the 3D cultures on microcarriers, a monolayer of CoO 0% and 2% PG and Synthemax II (Corning) microspheres were placed into low-adhesion 96-well plate (Costar^®^) in order to prevent cell attachment to the microwell surface. As the microspheres presented a different size distribution, surface area available for cell attachment was kept constant between the different microcarriers by adding different quantity of each composition (5 and 3.4 mg for PG and Synthemax II microspheres respectively). The microspheres were UV sterilised for 1 h 30 min. MSCs were seeded on the microcarriers monolayer at a seeding density of 5000 cells/well and then placed in the incubator at 37°C/5%CO_2_ for up to 14 days, with media replaced every 2 days.

Cell number was assessed at six different time points using a Quant-iT™ Picogreen^®^ dsDNA kit (Invitrogen). At each time point, triplicates of each condition were washed with 0.2 M carbonate buffer and lysed with 150 µL 0.1% Triton-X in 0.2 M carbonate buffer. Lysates were stored at −80°C until processing. On the day of the experiment, lysate samples were thawed at 37°C and pipetted up and down to fully lyse the cells. About 50 µL of sample were transferred to a 96 well plate. A standard solution of Lambda DNA was prepared and 50 µL of each dilution was transferred in triplicates to the 96 well plate. A working solution of the PicoGreen reagent was prepared by making a 1:50 dilution in TE buffer and 50 µL were added to each sample/standard. The plate was shaken and incubated in the dark for 5 min at RT. The plate was read at 485 nm emission and 538 nm emission using a fluorescent plate reader (Synergy HT, BioTek). Two independent experiments were performed using two different donors, six replicates per condition.

### Immunocytochemistry

Samples from the 2D (conditioned media) and 3D (microspheres) experimental groups were tested for extracellular matrix (ECM) protein expression through immunocytochemistry. Samples from the 2D and 3D group were fixed at day 7 post seeding and day 10 or 14, respectively. In both experimental groups, cells were fixed in 4% PFA (in PBS) for 20 min, rinsed with PBS (3×) and blocked in 3% BSA for 1 h at RT. Samples were then incubated with the following staining solution (in 3% BSA): 1/200 type I collagen (Abcam), 1/200 fibronectin (Abcam), 1/100 osteopontin (Abcam) and 1/100 osteocalcin (BD Biosciences). Primary antibody incubation was performed overnight at 4°C.

HIF1α nuclear staining was performed on the conditioned media group using 1/200 antihuman HIF1α (BD Biosciences) in PBS with 0.2% Triton X-100 and 10% goat serum, and kept at 4°C overnight as previously described.^37^

After PBS rinsing (3×), secondary antibody incubation was performed for 1 h at RT in the dark using the following staining solution (in 3% BSA): 1/300 Alexa Fluor 488 (Life Technologies) and 1/300 Alexa Fluor 594 (Life Technologies). This was followed by nuclear staining with 1/1000 Hoechst 33342 (Life Technologies) for 5 min at RT. Wells were then rinsed and stored in PBS until imaging. All fluorescent samples were analysed using a LSM Zeiss 710 confocal microscope and an Evos Fluorescent Cell Imaging System (Thermo Scientific) with 5×, 10× and 20× objectives.

### Alkaline phosphatase and Alizarin Red staining

Samples from the 2D (conditioned media) group were tested for alkaline phosphatase activity and Alizarin Red (AR) staining. Alkaline phosphatase activity was determined by incubating the cells for 5 min in 1 mg/mL Fast Red-TR Salt (Sigma) and 0.2 mg/mL Naphthol AS-MX phosphate (Sigma) in 0.1 M Tris-HCl, pH 9.2.

For AR, samples were fixed in 4% PFA (in PBS) for 20 min at RT and rinsed with deionised water (×3). Staining with 40 mM AR (in dH_2_O, pH 4.2) was performed for 1 h. Samples were then rinsed with deionised water to remove excess stain.

### RNA isolation and cDNA synthesis

For RT-PCR, a monolayer of CoO 0%, 2% and Synthemax II microspheres was placed in 24 ultra-low attachment plates (Costar^®^) and UV sterilised for 1 h 30 min. hMSCs were seeded at a density of 30,000 cells/well. The following time point were analysed: day 4, 7 and 14. Three independent experiments were performed using three different hMSCs donors.

RNA isolation and cDNA synthesis Total RNA was isolated using an RNeasy Mini Kit with on-column DNase treatment (QIAGEN VWR) according to the protocol given by the manufacturer. The concentration and purity of RNA was determined by using a NanoDrop spectrophotometer (NanoDrop Technologies). cDNA was synthesised from 100 ng of RNA using SuperScript III First-Strand Synthesis SuperMix (Invitrogen, Life Technologies) in a total volume of 21 μL, as per manufacturer’s instructions. An equivalent volume of DNase and RNase-free water (Sigma) was used in place of RT Enzyme Mix for no-RT controls.

### Quantitative real-time polymerase chain reaction (qPCR)

qPCR reactions were set up in triplicates with each reaction having a total volume of 10 μL containing 1X Platinum SYBR Green qPCR SuperMix-UGD (Invitrogen), 0.2 μM forward and reverse primers and 1 μL cDNA. A CFX Connect™ Real-Time PCR Detection System (Bio-rad, USA) was used at standard cycling parameters of 50°C for 2 min, 95°C for 2 min and then 95°C for 15 s and 60°C for 30 s for a total of 40 cycles. qPCR data were analysed using the delta delta method (ΔΔCt) using GAPDH as the reference gene and expression in the Synthemax II microcarriers as control.

### Human VEGF ELISA

A Quantikine^®^ ELISA assay (R&D Systems) was used to detect VEGF level in cell culture supernatant. Cell culture supernatant was harvested at day 1 and 7 from hMSCs cultured on CoO 0%, 2% and Synthemax II microspheres in low attachment 96 well plates. Cell culture supernatant was collected from two separate experiments, performed using two different donors. Samples of cell culture supernatant were centrifuged at 13,000 rpm for 20 min at 4°C and stored at −80°until processing. The ELISA assay was performed according to manufacturer instructions and normalised per DNA content, as quantified by Picogreen^®^.

### In vitro vascularisation assay

The day before the start of the vascularisation assay, HUVECs media EGM™-2 was removed and replaces with EBM™-2 in the absence of growth factors.

A Growth Factor Reduced (GFR) Matrigel^®^ (Corning) was used as a substrate for endothelial cells attachment. About 55 µL of Matrigel solution was added to wells of a precooled 96 well plate and incubated at 37°C for 1 h. HUVECs were seeded at a density of 15,000 cells/well in 75 µL of EBM (Lonza). Conditioned media from PG microspheres were obtained as previously described. The effect of conditioned media from the microspheres was tested in three independent experiments, with four replicates per condition. Wells supplemented with EGM2 media were used as negative control.

### Ex vivo CAM assay

Fertilised White Leghorn chicken eggs were incubated at 37°C in a humidified atmosphere (at >60% relative humidity). After 3 days incubation, 2 to 3 mL of albumin was withdrawn, using a 21 gauge needle, through a small opening at the large blunt edge of the egg to minimise adhesion of the shell membrane with CAM. A square window of 1 cm^2^ was opened in the egg shell and sealed with transparent adhesive tape to prevent dehydration. The eggs were returned for further incubation.

On embryonic day 8, the following implants were placed on top of the CAM:

(1) clusters of equivalent size of hMSCs cultured on CoO 0% and 2% microspheres for 7 days.
(2) equivalent volumes of CoO 0%, CoO 2% and Synth II microspheres embedded in 35 µL type I collagen gel (BD, Bioscience), produced according to manufacturer instruction.

After implantation, eggs window where sealed with transparent adhesive tape and returned to the incubator. Images were taken 7 days post implantation and samples of CAM containing the implant material were harvested and fixed in 4% PFA for 1 h at RT.

### Microfluidic culture device

#### Fabrication and assembly

The microfluidic culture device 2-layer layout was drawn using the software Layout Editor and printed onto HY2 glass plates (Konica Minolta) using a photoplotter (MIVA Xenon Photoplotter, MIVA Technologies). Devices features were formed by SU-8 2100 (Microchem) photolithography. Feature height and integrity were confirmed by optical surface profilometry (Veeco NT1100, Plainview). SU-8 device masters were silanised with chlorotrimethylsilane (CTMS) to facilitate easy removal of the moulded device. Devices were then formed with standard soft lithography techniques with poly(dimethylsiloxane) (PDMS) (Sylgard 184, Dow Corning), using a 10:1 mixture of silicon elastomer and curing agent. The mixture was then degassed under vacuum and poured onto the master and cured at 65°C for 2 h. After cutting, chambers in the bottom layer were then filled with microspheres. Input and output ports were obtained on the top layer (channel) through a 0.5 mm biopsy puncher. Device layers were bonded with O_2_ plasma (Harrick Plasma, 20 s, 10 W, 380 mTorr O2), with the channel PDMS layer aligned on top of the bottom layer.

The device was then left overnight at 95°C, before being submerged in 70% ethanol under vacuum, to be gradually filled with liquid, preventing bubble trapping, as described by Monahan et al.^38^ The device was then perfused by connecting the inlet to a syringe pump, through external connections made of tygon tubing and stainless steel couplers (Linton Instrumentations).

Equal distribution of microspheres throughout the device was tested by analysing images of each chambers via ImageJ. The number of microspheres was found to be ~1450 per chamber, corresponding to ~35,000 per microfluidic device that is equivalent to the number of microcarriers necessary to cover the area of a well in a 24 wells plate.

#### Cell culture and ICC

Upon reaching confluency, hMSCs were harvested and resuspended at a density of 1 × 10^6^/mL in media. Cells were then injected using a 1 mL syringe within the microfluidic device and allowed to settle within the chambers. The device was then washed with fresh media in order to remove cells from the channels. The device was then placed in an incubator at 37°C/5% CO_2_ overnight before starting perfusion, in order to allow the cells to attach to the microspheres. If not differently stated, the media flow rate was kept to 20 µL/h and cells were cultured within the device for up to 7 days.

For the hMSC-endothelial cell interactions experiment, HUVECs (P5) were tagged with a green fluorescent CellTracker (Thermofisher) according to manufacturer instructions, trypsinised and resuspended at a density of 1 × 10^6^ cells/mL. The HUVEC suspension was then perfused within bioreactors pre-seeded with 7 day hMSC-microsphere cultures. The bioreactor was then perfused with a 50:50 DMEM/EGM-2 media solution. After 24 h, one group of reactors was fixed, after removal of unattached cells by perfusion with PBS. In a second experimental group, cells were left in co-culture for 48 h, in 50:50 DMEM/EGM-2 media, at a perfusion rate of 20 µL/h.

For imaging, all bioreactors were fixed in 4% PFA for 20 min at RT, blocked in 3% BSA for 1 h at RT and labelling with either collagen type I or extracellular fibronectin (1/200) was performed overnight at 4°C. Secondary antibody (1/300) was applied for 1 h at RT and nuclei were stained using a Hoechst solution (1/1000) for 5 min at RT. Images were then obtained using a confocal microscope and mean fluorescent intensity was quantified using ImageJ.

### Statistical analysis

All data are shown as mean ± standard deviation, if not differently stated. Statistical signicance was assessed via one-way ANOVA, followed by Tukey’s multiple comparison test (significance level ⩽0.05) or two-way ANOVA followed by Bonferroni’s multiple comparison test (significance level ⩽0.05), using GraphPad Prism software (GraphPad,), as reported per experiment.

## Results

### hMSC population of PG microspheres and formation of tissue-like aggregates

**Figure 1.**
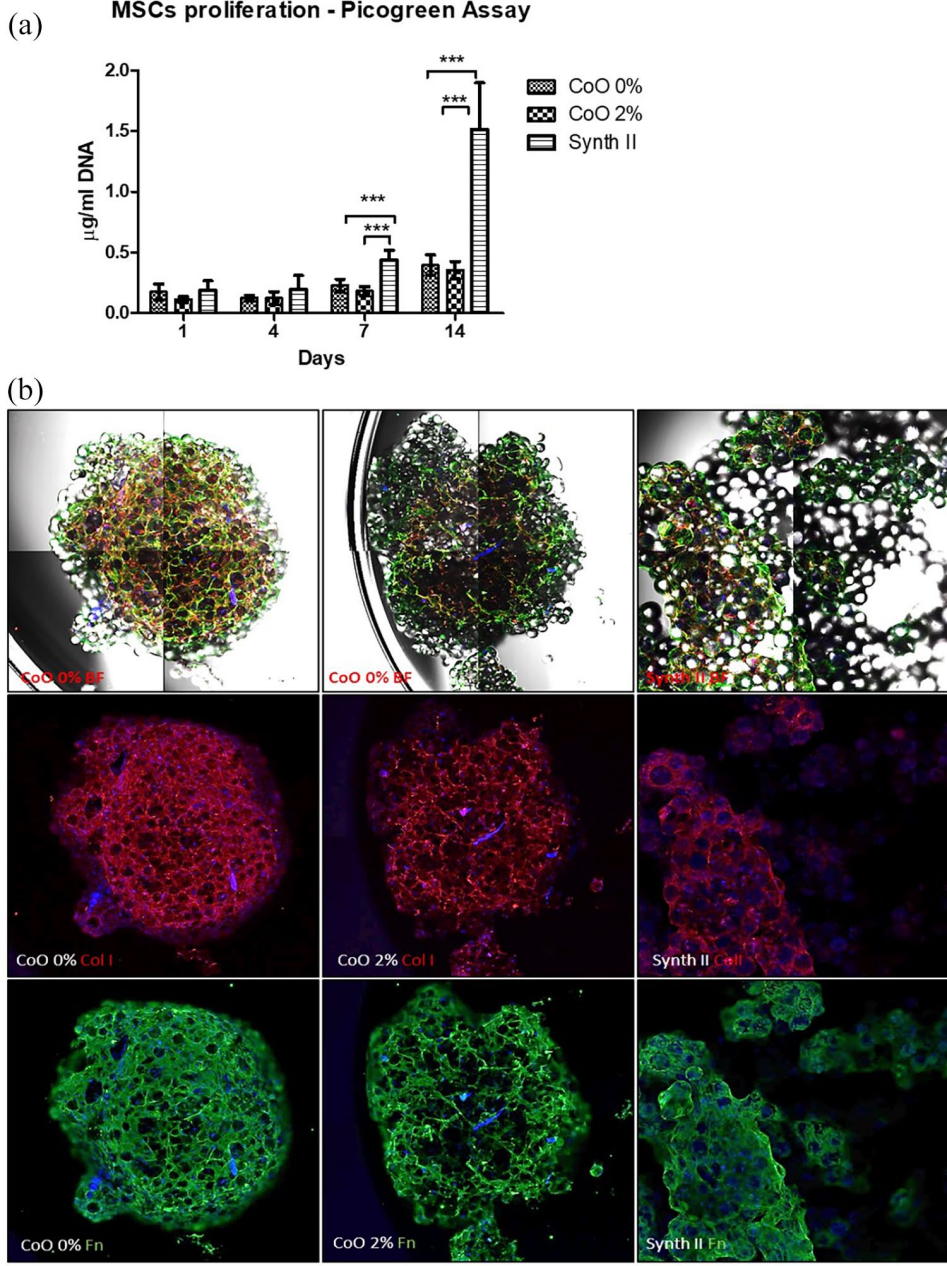
Creation of engineered tissue clusters from hMSCs and PG microspheres. Proliferation of hMSCs on monolayers of CoO 0%, 2% and Synthemax II microspheres was monitored over 14 days and morphology of the subsequent structure was assessed, along with extracellular matrix deposition. Indirect measurement of cell number via DNA quantification suggested that cell number was lower on PG microspheres at day 7 and 14, compared to that on Synthemax II (a). Morphologic analysis revealed that cells on the PG microspheres formed tight clusters rich in type I collagen and fibronectin, whereas on Synthemax II clustering was minimised and remained predominantly as a monolayer (b). Data show pooled results obtained from two different donors, *n* = 6/donor. Data are shown as Mean ± SD, statistically compared by a two-way ANOVA followed by Bonferroni post test. ***p < 0.01. (Fibronectin = green; type I collagen = red; DAPI = blue). Images were obtained using a ×4 objective.

Monolayers of microspheres (CoO 0%, CoO 2% and Synthemax II) were seeded with hMSCs and cultured for 14 days. Cell number was measured indirectly as a function of amount of DNA present and the resulting culture was assessed morphologically (Figure 1). At day 1, there was no significant difference in DNA levels between microspheres, suggesting similar level of cell attachment to all microspheres. At day 7 and 14 post-seeding, DNA content from cells on PG microspheres was found to be significantly lower than from the Synthemax II microspheres (Figure 1(a)). Bright field and confocal microscopy analysis revealed that whilst extracellular deposition of type I collagen (Red) and fibronectin (Green) by hMSCs was evident across all three microspheres, the Synthemax remained predominantly as a monolayer, whereas hMSC-PG microspheres self-assembled into tissue-like clusters (Figure 1(b)). After 2 weeks in culture, both type I collagen and fibronectin were secreted and distributed on the CoO 0% scaffold. Both proteins were also expressed on the CoO 2% microspheres, however their secretion was found to be less uniformly distributed throughout the construct. Extracellular expression of type I collagen and fibronectin was also clearly visible on the Synthemax II microspheres.

### Osteogenic differentiation of hMSCs within engineered tissue-like aggregates

**Figure 2.**
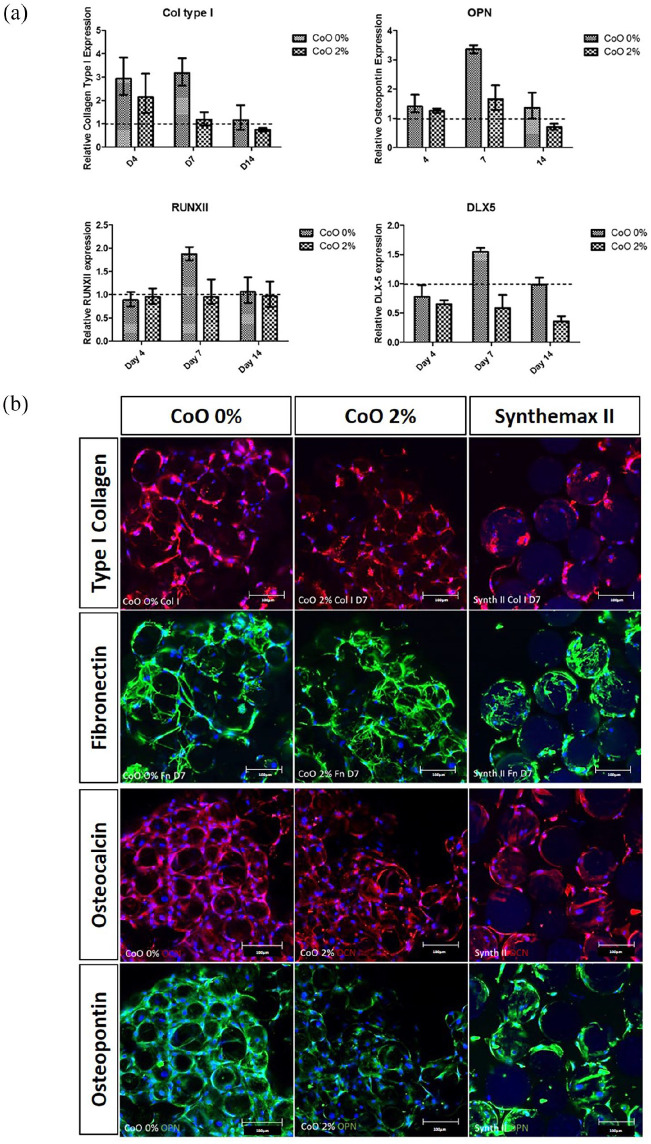
Osteogenic differentiation of hMSCs within engineered tissue-like aggregates. Quantitative real-time PCR analysis of osteogenic gene expression revealed that gene expression was selectively upregulated on CoO 0% PG in comparison to Synthemax II in a time-dependent fashion (a). COL1 upregulation was detected from day 4, whereas RUNX2, OPN and DLX5 were upregulated at day 7. Expression of these genes on CoO 0% microspheres was consistently higher than on CoO 2% surfaces. Immunofluorescence labelling of type I collagen, fibronectin, osteocalcin and osteopontin at day 7 (b) revealed that all microsphere materials supported osteogenic differentiation. Labelling appeared greater on the CoO 0% microspheres compared to CoO 2% for type I collagen, osteocalcin and osteopontin. Images are representative of the trend in expression observed in two independent experiments performed using different hMSCs donors. Scale bars = 100 µm.

Osteogenic differentiation in response to the PG microspheres was characterised by assessing transcription of key genes associated with osteogenic differentiation and then normalised to gene expression in response to Synthemax II (Figure 2(a)). Quantitative real-time PCR revealed that type I collagen, whose elevated production is an early event in osteogenic differentiation, was upregulated in response to CoO 0% PG by 3-fold compared with Synthemax II at days 4 and 7. RUNX2, OPN and DLX5 were all upregulated on CoO 0% PG microspheres at day 7 only. For all genes, the same level of upregulation was not observed for CoO 2%, indeed it was mostly absent.

Next, protein markers of osteogenic differentiation were detected by immunofluorescence labelling of type I collagen, fibronectin, osteocalcin and osteopontin at day 7 (Figure 2(b)). Images revealed that all microsphere materials supported expression of proteins associated with osteogenic differentiation. Clear evidence of clustering was seen on the PG microspheres, as the labelling formed a continuous mesh for both CoO 0% and CoO 2% conditions. Labelling intensity and frequency appeared greater on the CoO 0% microsphere material than on CoO 2% for type I collagen, osteocalcin and osteopontin. This was consistent with the elevated expression of osteogenic genes observed with PCR. The Synthemax II microspheres remained largely as a monolayer, with discrete labelling around individual microspheres.

### Osteogenic potential of soluble species released from PG microspheres

**Figure 3.**
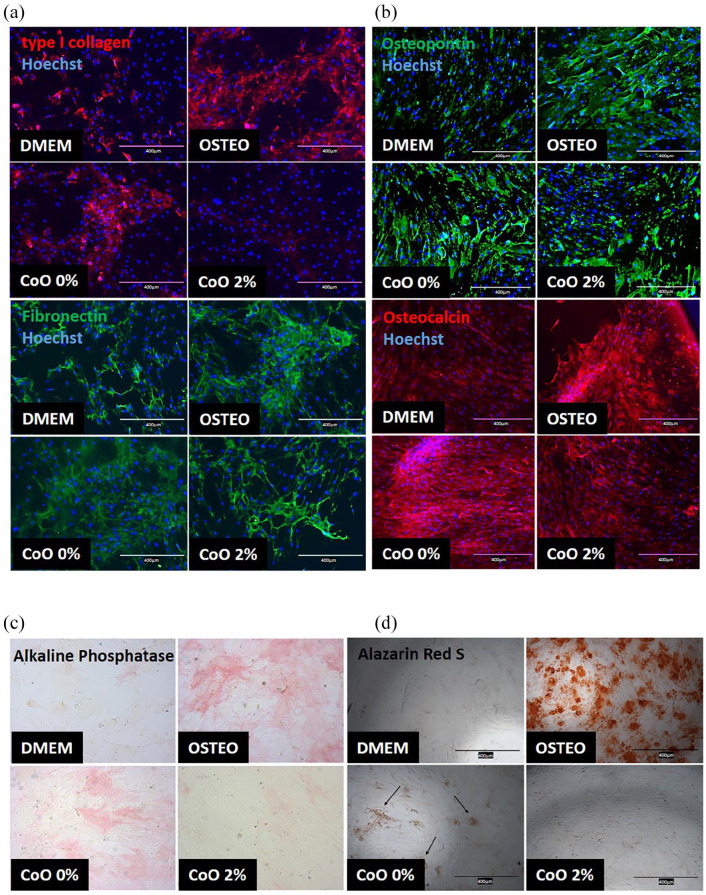
Influence of soluble species released from PG microspheres on osteogenic differentiation of hMSCs. (a and b) Immunofluorescence labelling of hMSC cultures for osteogenic differentiation marker expression in response to conditioned media from PG microspheres, compared with standard DMEM (negative control) or osteogenic media (positive control). Analysis of (a) type I collagen and fibronectin after 7 days culture and (b) osteopontin and osteocalcin after 10 days culture revealed that CoO 0% microsphere-conditioned media induced expression patterns similar to the osteogenic media condition, whereas conditioned media from CoO 2% microspheres did not. (c) Alkaline phosphatase activity followed a similar pattern, as did Alizarin Red S (d), although for the latter, the staining was not as prominent as for the osteogenic control, suggesting fewer calcium deposits. Images are representative of the trend in expression observed in two independent experiments performed using different hMSCs donors. Scale bars = 400 µm.

To determine whether the effects of CoO 0% and CoO 2% PG microspheres were a result of soluble factors released from those materials, microspheres were incubated in standard DMEM media for 24 h and then the effect of resulting conditioned media on various osteogenic responses was assessed. hMSCs were also cultured in standard DMEM media (negative control) or in osteogenic media (positive control). Soluble species released from CoO 0% PG microspheres induced deposition of type I collagen and fibronectin after 7 days to similar levels as seen for osteogenic media (Figure 3(a)). Similarly, production of osteopontin and osteocalcin in response to CoO 0% PG microsphere-conditioned media after 10 days was also similar to the osteogenic media positive control (Figure 3(b)). By contrast, hMSCs cultured with CoO 2% PG microsphere-conditioned media responded more similar to the negative control. A similar pattern for alkaline phosphatase activity after 10 days was revealed for the PG microsphere-conditioned media (Figure 3(c)). CoO 0% PG-conditioned media induced alkaline phosphatase activity similar to the osteogenic media, whereas CoO 2% PG-conditioned media promoted only weak staining similar to the standard DMEM negative control. For Alizarin Red staining performed after 10 days, CoO 0% PG microsphere-conditioned media induced only weak staining compared to the osteogenic media positive control. However, this was still visibly higher than for the CoO 2% condition (Figure 3(d)).

### Vascular response of hMSCs to PG microspheres

**Figure 4.**
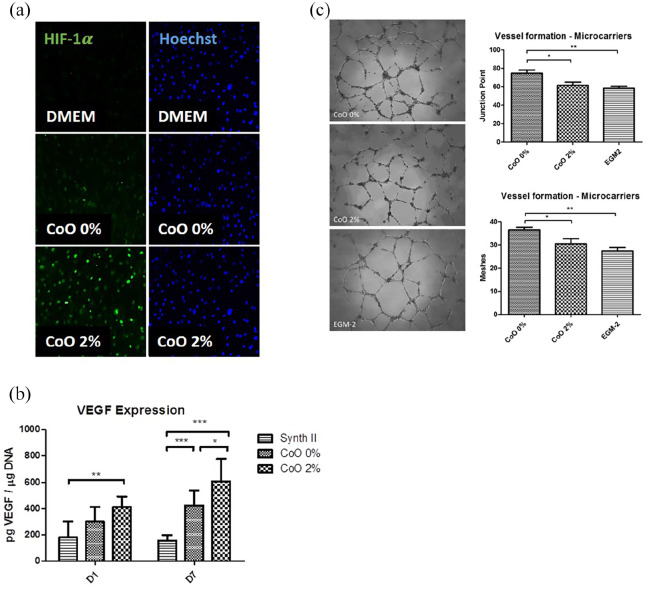
Vascular response of hMSCs to PG microspheres. (a) Immunofluorescence labelling of nuclear HIF1α (green) by hMSCs in response to PG microsphere-conditioned media revealed that CoO 2% induced strong and widespread expression of HIF1α whereas CoO 0% induced little, very weak expression. HIF1α was completely absent when cells were cultured in standard DMEM media. (b) VEGF levels in cell culture supernatant from hMSCs grown on CoO 0%, CoO 2% and Synthemax II microspheres was measured at day 1 and day 7 from seeding. VEGF production was consistently elevated in response to CoO 2% compared with Synthemax II (day 1 and 7) and CoO 0% (day 7). (c) Endothelial tubule formation on Matrigel was assessed in response to media conditioned by CoO 0% and 2% microspheres. After 18 h, increased tubule formation was observed when HUVECs were grown with CoO 0%-conditioned media, resulting in enhanced junction point and meshes formation. CoO 2% performed similarly to the positive control in terms of junction points number. However, from a visual analysis, tubule formed appear less developed and robust in the presence of cobalt ions. Data in (b) was from two independent experiments, performed using different donors, in triplicate. Data are shown as Mean ± SD, statistically compared using a two-way ANOVA followed by Bonferroni’s post test. Data in (C) was from three independent experiments (*n* = 4/experiment) and are shown as Mean ± SD and statistically compared by a one-way ANOVA followed by Tukey’s multiple comparable test *p < 0.05, **p < 0.01, ***p < 0.001.

In the next series of experiments, we wanted to understand whether the reduced osteogenic potential of CoO 2% PG microspheres was mitigated by improved vascular support. We generated conditioned media from pre-incubating standard DMEM with CoO 0% and CoO 2% PG microspheres for 24 h and then incubated hMSCs with the conditioned media or standard DMEM culture media for 7 to 10 days. We then performed immunofluorescent labelling against HIF1α and found that CoO 0% PG microsphere-conditioned media induced low levels of HIF1α whereas CoO 2% induced very high levels of HIF1α (Figure 4(a)). We then assessed VEGF production by hMSCs (Figure 4(b)) and found that whilst both materials released soluble species that promoted VEGF release, the results were most prominent for CoO 2% microspheres, with early effects at day 1 (*p* < 0.01 vs Synthemax II control) and maintained higher levels at day 7 (*p* < 0.001 vs Synthemax II and *p* < 0.05 vs CoO 0%). Next, the functional effect of PG microsphere-conditioned media on endothelial tubule formation was assessed (Figure 4(c)). HUVECs were seeded onto Matrigel and incubated with either growth factor-enriched endothelial media (EGM-2) as a positive control, or with the conditioned media for 18 h. Surprisingly, whilst CoO 2% PG microsphere-conditioned media induced branchpoint and mesh formation similar to the positive control, the CoO 0% PG microsphere-conditioned media induced significantly higher branchpoints (*p* < 0.01 vs EGM-2 and *p* < 0.05 vs CoO 2%) and mesh formation (*p* < 0.01 vs EGM-2 and *p* < 0.05 vs CoO 2%).

### Ex vivo vascularisation of PG microspheres and hMSC-PG microsphere aggregates

**Figure 5.**
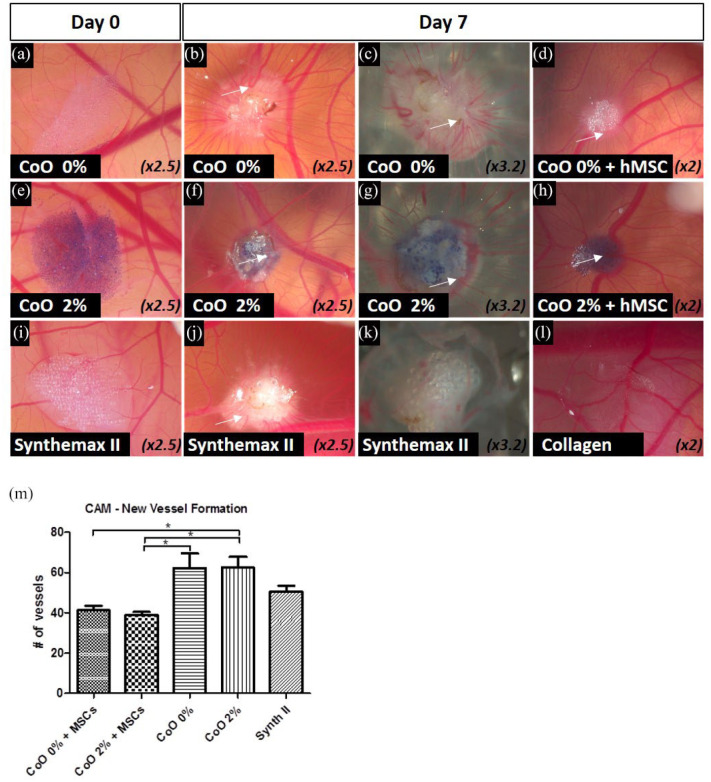
Ex vivo vascularisation in a chick embryo chorioallantoic membrane (CAM) assay. (a–l) Images capturing the vascular response of the chick CAM to CoO 0% PG and CoO 2% PG mircospheres with or without hMSCs, along with Synthemax control. Evidence of vascular loops was seen at 7 days for all microsphere and cell-microsphere conditions (examples indicated by arrows). (m) Quantification of vessel ingrowth indicated that hMSC-PG microsphere material was less vascularised than cell-free scaffolds. Highest number of new formed vessels was observed on cell-free CoO 0% and 2% microspheres, although this was not found to be significant compared to the Synthemax II. Data are presented as Mean ± SD, *n* = 4–8, and statistically compared by a one-way ANOVA followed by Tukey’s multiple comparison test, *p < 0.05.

A chick embryo chorioallantoic membrane (CAM) assay was used to determine whether vascularisation of implanted PG microspheres with or without hMSCs was achieved. hMSCs were cultured on PG microspheres for 7 days before implantation in the CAM. Cell-free microspheres were embedded in a type I collagen gel before implantation. All implants were harvested after 7 days from the day of implantation. Cell-free microcarriers appeared as a monolayer on the CAM surface at the day of implantation (Figure 5(a), (e) and (i)). On the harvesting day, all microspheres were found to cluster together in a single mass, with evidence of vessel ingrowth (Figure 5(b), (f) and (j)) and vascular loops (examples indicated by arrows). Patches of collagen gel without microcarriers were also implanted as a negative control and there appeared to be little vascularisation. hMSC-PG microsphere clusters were also found to be successfully vascularised at the end of the incubation time (Figure 5(d) and (h)). However, subsequent quantification revealed that the hMSC-PG microsphere clusters did not promote vascularisation as well as the PG microspheres on their own (Figure 5(m)).

### Application of a controlled perfusion microfluidic platform for formation and characterisation of self-assembled hMSC-PG microsphere structures

**Figure 6.**
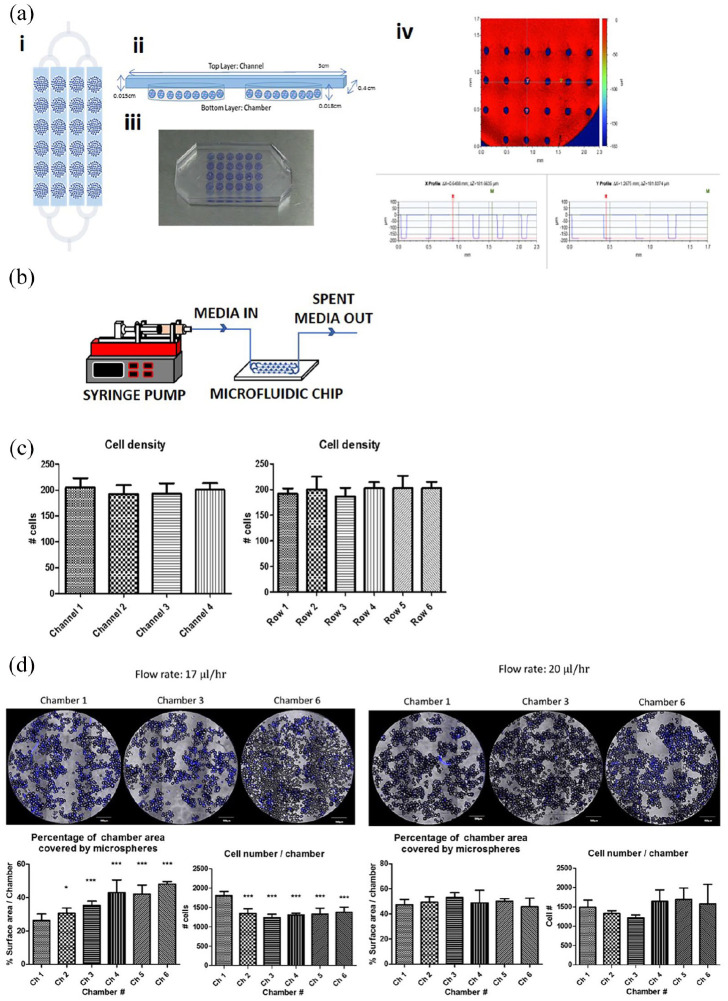
Microfluidic device for characterising hMSC-PG microsphere interactions. (a) Microfluidic bioreactor schematic and optical surface profilometry. (i) Plan view of the microfluidic bioreactor, showing a single inlet, branching to four channels that feed parallel arrays of six serially connected culture chambers. (ii) Side view depicting relative position of chambers in the bottom layer of PDMS and the perfusion channels sitting in the upper layer. Here, the illustration shows two serial chambers filled with microspheres, interconnected by a perfusion channel. (iii) Photograph of the PDMS microfluidic culture device, where each chamber is filled with CoO 2%-doped PG microspheres (blue/purple colour). (iv) Optical surface profilometry of a chamber, showing the depth of the chamber at two different points x and y; a depth of ~180 µm was confirmed and the presence of posts is shown in blue. (b) Schematic of experimental setup, where programmable syringe pump was used to perfuse the contents of the microfluidic chip with fresh culture medium. (c) Monitoring of cell distribution across the parallel channels and across the rows revealed even cell seeding. Hoechst-stained nuclei from one random × 10 field from each chamber was quantified across three independent experiments. (d) Assessment of media flowrate indicated that 17 µL/h was insufficient to maintain hMSC survival downstream of chamber 1 and the clustering of cell-microsphere material was diminished, resulting in increased surface area covering from chamber 2 onwards. Raising the flowrate to 20 µL/h led to consistent cell yields and even clustering across all chambers in the sequence. For (d) data were pooled from two independent bioreactors (using different hMSCs donors) and are shown as Mean ± SD, *N* = 3–4. Representative images for each experiment are shown. Scale bars: 500 µm. Statistical significance was assessed via a two-way ANOVA followed by Bonferroni post test.

A microfluidic bioreactor was fabricated from PDMS using soft-lithography techniques (Figure 6) in order to monitor cell material interactions more accurately in a closed, perfused system. This was done to ensure results were not subjected to the compound effects of manual operation and manipulation of material, which could potentially disrupt cell-material clusters. The culture device comprises two layers: the upper layer that houses the perfusion channels and the bottom layer that houses the culture chambers. The device, filled with CoO 2% PG microspheres can be seen in Figure 6(a), iii. Optical surface profilometry revealed that chambers had a depth of ~180 µm and micropillars were present (Figure 6(a), iv). Microcarriers were pre-seeded and after plasma bonding of the upper layer, cells were seeded into the device and then perfused with media using a programmable syringe pump (Figure 6(b)). Cell number was quantified across all chambers and rows after hMSC seeding in the bioreactors (Figure 6(c)). Cell nuclei were labelled with Hoechst and images of one random field (×10) of each chamber were taken from three independent experiments. No variation in cell number was observed, thus uniform cell distribution across the reactor was achieved. Next, determination of optimal flowrate on hMSCs expansion within the microfluidic bioreactor was assessed using two donors (Figure 6(d)). A flow rate of 17 µL/h was adopted, based on the workings of Young and Beebe^39^ and Titmarsh et al.,^40^ where flow rate is calculated as a function of the effective culture time (ECT) based on media exchange frequency at the macroscale. It was found that cell survival over a 7 day culture period dramatically decreased from the first chamber to the subsequent chambers in the reactor (*p* < 0.001). Furthermore, cell clustering, as shown by microscopy, was inhibited and the presence of cell-free microspheres was evident on the last row of chambers. By minimally increasing the flowrate to 20 µL/h, cell number was found to increase in the second half of the bioreactor; however, this effect was not significant (*p* > 0.05), confirming a condition that supported cell viability across all chambers. Similar level of clustering was observed in all chambers (*p* > 0.05).

### Characterisation of hMSC-PG microsphere interactions using a controlled perfusion microfluidic platform

**Figure 7.**
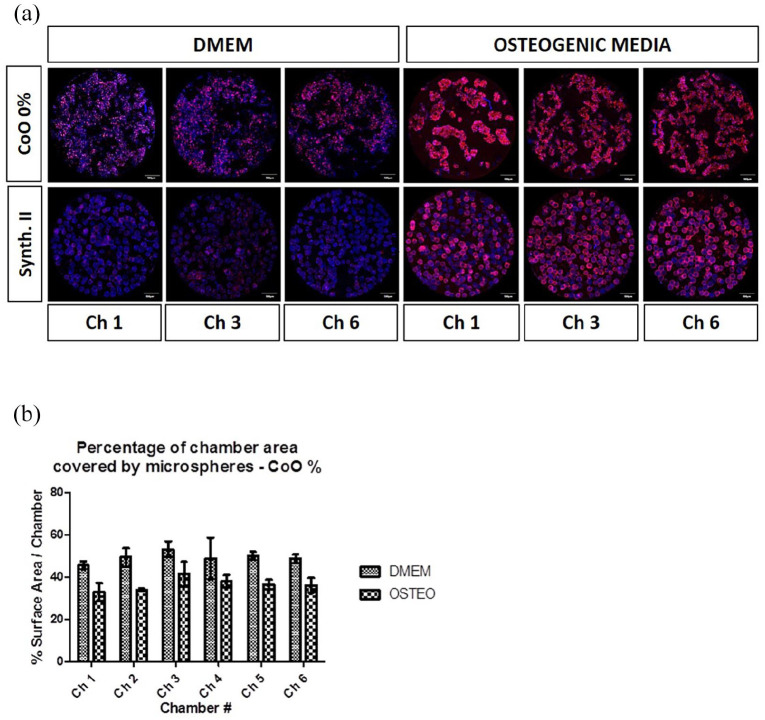
Characterisation of hMSC-PG microsphere behaviour in absence of manual manipulation procedures. (a) Validation of the microfluidic device and further characterisation of cell-material interactions without manual manipulations was achieved by culturing hMSCs on CoO 0% PG microspheres side-by-side with the Synthemax II control in the presence of either standard DMEM media or osteogenic media. Osteogenic media induced type I collagen production on both surfaces but clustering into tissue-like structures was only observed for CoO 0% materials. The CoO 0% microsphere material was also able to upregulate type I collagen production independent of chemical cues. (b) Assessment of cell-material clustering on CoO 0% into tissue-like structures was indirectly calculated by measuring surface area coverage, which was reduced in the presence of osteogenic media. The experiments was performed using two different donors, showing a similar trend. Confocal images obtained with one representative hMSC donor are shown in the figure, scale bars = 500 µm.

The microfluidic device was next used to characterise, in a closed and automated perfusion system, hMSCs responses on CoO 0% PG microspheres side-by-side with the Synthemax II control microspheres (Figure 7(a)). In standard DMEM-based culture media, expression of type I collagen was minimal, whereas in osteogenic culture media the signal intensity was very strong (Figure 7(a)). This was as expected from static culture results and enabled the device to be validated. Comparing between CoO 0% PG and Synthemax II microspheres, greater type I collagen expression was observed on CoO 0% PG. In DMEM conditions, type I collagen labelling was absent whereas on the PG microsphere a weak signal was seen. In osteogenic media a moderately strong signal was seen on Synthemax II but consistent with earlier observations (Figures 1 and 2) no clustering was observed. However, on CoO 0% PG microspheres, a strong signal and extensive clustering into tissue-like aggregates was seen. This reveals synergy between chemical cues and biomaterial cues in the self-assembly of tissue-like aggregates. This clustering was further characterised by calculating the percentage of the surface area covered by CoO 0% microspheres cultured with either standard DMEM or osteogenic media across two bioreactors seeded with two hMSC donors (Figure 7(b)). Across all chambers in the device a consistent trend of reduced surface area covered, indicating higher degree of clustering, compared with cells cultured on Synthemax. There did not appear to be variations in cell responses according whether they occupied wells near the media inlet versus those further downstream. This suggests that the effects of ion release from microcarriers is a stronger influence than paracrine effects from upstream wells.

### Characterisation of direct hMSC-endothelial cell interactions using a controlled perfusion microfluidic platform

**Figure 8.**
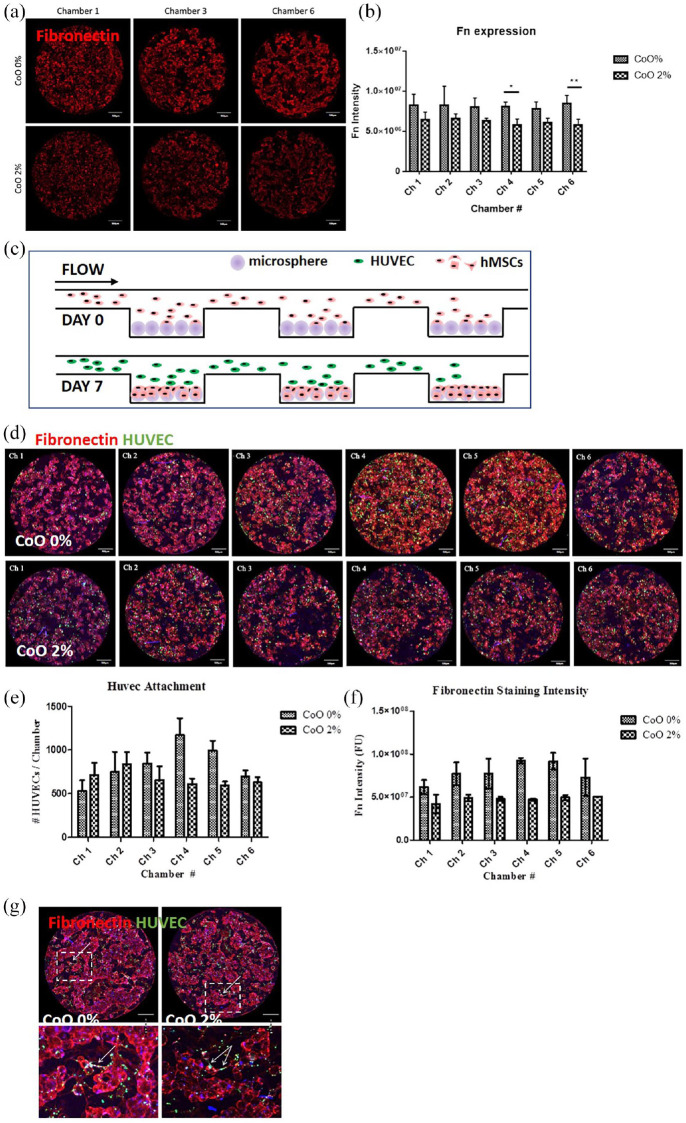
Characterisation of hMSC-endothelial cell interactions using the microfluidic device. (a) Extracellular fibronectin deposition by hMSCs was assessed after 7 days under perfusion and (b) a tendency of greater deposition in response to CoO 0% was observed. (c) Representative images showing HUVEC attachment to hMSC-microsphere material. Quantification indicated that (d) HUVEC attachment to 7 day hMSC-microsphere cultures after 24 h was increased on CoO 0% PG microspheres compared to CoO 2% PG microspheres in downstream chambers (4–5), associated with corresponding fibronectin deposition (e). (f) Extended culture of HUVECs on the hMSC-microspheres for a further 48 h (to a total of 3 days culture) revealed interactions between HUVECs and the hMSC-microsphere clusters including limited formation of tubule-like structures (indicated by arrows). For (b) Mean ± SD, *n* = 4; For (d and e), pooled data from two independent experiments are shown as Mean ± SD, *n* = 2. Scale bars = 500 µm.

In the next experiment, we again utilised the microfluidic culture device to better understand how the PG microsphere materials might influence the interaction between hMSCs and HUVECs (Figure 8). First, we assessed extracellular fibronectin deposition by hMSCs on the CoO 0% and CoO 2% PG microspheres. Fibronectin deposition by hMSCs showed a trend of consistently higher deposition in response to CoO 0% PG microspheres, although this was only statistically significant in two instances (Figure 8(a) and (b)). Next, we introduced HUVECs to the culture after hMSCs had been cultured for 7 days (Figure 8(c)). HUVEC attachment after 24 h was selectively higher where hMSCs were cultured on CoO 0% PG microspheres (Figure 8(d) and (e)) and this correlated with elevated fibronectin deposition in those cultures (Figure 8(f)). When assessing interaction of HUVECs with the hMSC-microsphere clusters after 3 days, there appeared to be more direct interaction between HUVECs and the fibronectin-rich matrix on CoO 0% microspheres than on CoO 2% microspheres (Figure 8(g)). Some occasional cellular alignment into tubule-like structures was also evident.

## Discussion

Cell culture microcarriers allow cell expansion in a 3D bioreactor environment^7^ and can also be used as a scaffold for in vivo implantation, if made from a biodegradable and biocompatible material.^5^ Our rationale for processing PG glasses into microspheres was to provide a flexible system for achieving scalable production of engineered bone-like tissue that could ultimately be adapted to individualised defect shapes and geometries. Chemically-coated polystyrene microcarriers (Synthemax II) were used as a control, as these microcarriers have been previously reported to support hMSCs proliferation while retaining tri-lineage differentiation potential for multiple passages.^41^ Even though hMSC expansion was much lower on PG microspheres than on Synthemax II microcarriers, hMSCs on PG microspheres had a more natural tendency to cluster into tissue-like aggregates, with all microspheres being embedded in a single construct by 14 days. This was not the case for hMSCs on Synthemax II microcarriers, where aggregation started occurring only towards the end of the culture period. Of note, the diameter of the Synthemax II microcarriers was higher than the PG microspheres (168.5 and 85 µm, respectively), although being significantly less dense (1.03 g/cm^3^ compared to 2.65 g/cm^3^ of CoO 0% and 2.68 g/cm^3^ of CoO 2%). The size and density of the microspheres might have contributed to the different level of aggregation observed.

The results of this study confirmed a general trend of elevated osteogenic activity in response to CoO 0% PG glass microspheres compared to Synthemax II in the absence of chemically defined osteogenic media, revealed by both transcript levels and protein deposition (Figure 2). This was lacking for the CoO 2% material. Subsequently, a direct comparison of conditioned media from PG microspheres revealed that soluble species from CoO 0% PG microspheres induced osteogenic protein expression patterns more similar to chemically defined osteogenic media (Figure 3). On the other hand, soluble species from CoO 2% PG microcarriers induced expression patterns more similar to the standard culture media control. In a previous study, we characterised the release of Co^2+^ ions from CoO 0% and CoO 2% microspheres over a period of 21 days.^35^ Although CoO concentrations used in this study were lower than those recognised to cause cytotoxic effects on human osteoblastic cells^15,42^ and mesenchymal stem cells,^16^ these results suggest that the soluble divalent Co^2+^ ions may still interfere with Ca^2+^and Ti^4+^ signalling that usually (as in the case of CoO 0% microspheres) leads to molecular events, such as increased expression of osteogenic transcription factors and subsequent osteogenic protein.^14,43,44^

The observed lack of osteogenic induction on CoO 2% PG microspheres was expected to be somewhat compensated by clear improvements in vascular responses, an important consideration for creating 3D tissues that will require perfusion. The rationale for the inclusion of cobalt in these microspheres was based on the fact that cobalt has been shown to mimic hypoxia (in terms of response) and promote, for example, upregulation of HIF1α and VEGF expression,^16,17,25^ which in turn has been shown to lead to transient improvement of endothelial tubule formation.^25^ However, our data suggests that such expected functional improvements are not fully supported, and in fact, others have reported that vessel formation by endothelial cells exposed to cobalt ions was impaired, even though HIF1α was upregulated.^45^ After pre-conditioning basal EGM-2 media with PG microspheres for 7 days at 37°C, we observed a clear upregulation of HIF1α in hMSCs in response to CoO 2% PG microsphere-conditioned media, as opposed to CoO 0% (Figure 4(a)). Furthermore, VEGF secretion from hMSCs was consistenly higher in the presence of cobalt, compared to the control groups, thus confirming Co^2+^ ions release from the PG microspheres to be within functional range. However, when we tested functional tubule formation from endothelial cells in complete EGM-2 made from media conditioned with CoO 2%, we observed no differences to the complete non-conditioned EGM-2 positive control. Interestingly, conditioning from CoO 0% PG microcarriers resulted in significantly increased tubule formation (Figure 4(c)). This improvement after incubation with CoO 0% PG microspheres may also reflect increased Ca^2+^ availability for endothelial cell vascular responses, that is diminished in the presence of competing Co^2+^ as previously shown by our group.^15^ Indeed, Ca^2+^ signalling is critical for endothelial cell responses and reduced bioactivity due to competing Co^2+^ might counter any positive effects elicited by HIF1α-VEGF signal axis.

The final experiment that we performed to assess angiogenesis was the chick chorioamniotic membrane assay, whereby PG microspheres, with or without hMSCs, were grafted into the CAM to determine biocompatibility and to understand whether cobalt-doped PG microspheres would enhance angiogenic ingrowth. We logically assumed that hMSC-populated CoO 2% PG microspheres would promote the greatest angiogenesis response due to increased HIF1α and VEGF expression by hMSCs in the presence of CoO 2% that we saw in our earlier experiment (Figure 4(a) and (b)). However, there was no difference in angiogenic responses between hMSCs on CoO 0% or CoO 2% microspheres (Figure 5). What was even more striking was the fact that cell-free PG microspheres induced significantly greater angiogenic ingrowth than those loaded with hMSCs, but again with no distinction in responses where cobalt was present. In the current experiment we captured data after a 7 day period and it is possible that any subtle differences in rate of angiogenesis are only evident earlier. This would align with in vitro observations by Quinlan who reported longer and more mature endothelial cell-derived tubules on Matrigel at 4 and 12 h post-seeding in the presence of Co^2+^ but by 24 h there was no difference to the Co-free control.^25^ Therefore, the *window* in which any observable differences in rate of angiogenesis might be earlier. We had assumed moreover that the presence of hMSCs would allow continuous production of growth factors, simulating a more *physiologic* interaction with the CAM vasculature. However, the lower level of vascularisation of this group might be explained by the structure of the cell-loaded scaffold, as this appeared as a solid cluster embedded in a thick ECM. On the other hand, cell-free microspheres where seeded as a monolayer on the CAM surface thus potentially facilitating vessel ingrowth between the particles. This is of particular relevance from a tissue engineering perspective suggesting that control of construct size, shape and length of pre-implantation cell culture need to be carefully determined and controlled during in vitro modular tissue assembly.

The experiments described above clearly showed how working with cells and microspherical scaffolds is challenging both in terms of process robustness of the manual handling and also in relation to consistency of the subsequent characterisation that is achievable. Microfluidic bioreactors enable the relationship between process parameters such as channel size, flow rate, shear stress and cellular responses to be mapped simultaneously.^46,47^ The key advantage from a bioprocess discovery perspective is that multiple different microsphere substrates and nutrient feeds can be screened in parallel in a closed and semi-automated system in microliter quantities, enabling even the study of tissue formation within a well-defined microenvironment using minimum quantities of reagents. In terms of application of microfluidic bioreactors for creation of engineered bone, Lee and colleagues created multi-channel microfluidic devices to enable real-time monitoring of mineralised 3D tissue-like structures by osteoblasts.^48,49^ These devices were also used to visualise the complex interactions between osteoblasts with bacteria and antibiotics, and to perform reproducible co-culture experiments free from cross-contamination whilst using very small quantities of culture medium. Jusoh et al. have reported the development of a platform incorporating hydroxyapatite (HA) into a microfluidic chip as a mineralised bone tissue model for mimicking real bone angiogenesis.^50^ The formation of angiogenic networks was observed as a function of various HA concentrations.

We have previously designed and validated a microfluidic device platform to permit full factorial screening and monitoring of cells in 2D culture.^40^ The current device detailed in this work represents a substantial simplification of our previous design, whilst still leveraging the ability to establish and monitor multiple parallel cultures in situ. Furthermore, by the inclusion of wells into our current device, we were able to screen minute quantities of different cell-biomaterial combinations within a single perfused device, amounting to 24 discrete, serially-connected wells with a total working volume of >150 µL.

An optimal flow rate (i.e. 20 µL/h) required for culturing hMSC on PG microspheres within the microdevices was firstly identified. The use of the platform for screening experiments was validated via confirmation that a combination of CoO 0% PG microspheres and osteogenic media under perfusion resulted in the greatest degree of type I collagen deposition and cell-material clustering. We expected to observe paracrine mediated signalling influencing downstream wells in each series, based on our previous experience.^40^ However, this was not observed, likely due to the effect of high concentrations of soluble ions being released in close proximity to the cells overriding any secreted factor accumulation. Our observation of the strong effect of ions in 2D in the absence of any such carriers (Figure 3) support this. The fact that the microspheres can offer deterministic outcomes and override paracrine-mediated signalling is very exciting as it implies potential for better bone generation regardless of implantation site and the soluble factors present.

We then used the microfluidic device to examine how hMSCs and endothelial cells interact as this might give some clues as to why the elevation in HIF1α and VEGF expression in response to Co^2+^ does not translate to increased angiogenesis at the time observed. hMSCs were seeded onto the PG microspheres in the closed system and subjected to automated perfusion of culture media at 20 µL/h for 7 days, before endothelial cells were introduced. We found that fibronectin deposition was higher in the absence of cobalt and this translated to increased endothelial cell attachment in downstream wells. Fibronectin is an important extracellular matrix component for vascular development and enables provides a substrate for endothelial cell attachment via α5β1 integrins.^51^ Increased abundance of fibronectin deposition by hMSCs on CoO 0% PG microspheres, is therefore capable of promoting the attachment of endothelial cells.

## Conclusion

In conclusion, in this study we confirmed the suitability of titanium doped PG microspheres as a promising platform for scalable and customisable production of engineered bone. However, the functional responses of hMSCs to PG microspheres further doped with cobalt are not as anticipated and in fact, osteogenic and vascular responses characterised in vitro are more prominent in the absence of cobalt. Results obtained from the CAM assay underlined the importance of controlling the manufacturing of cellularised scaffold of defined size and shape to support in vivo vascularisation.

The development of a novel closed and semi-automated perfused culture device enabled us to gain insight as to how mesenchymal and endothelial cells interact on PG microsphere surfaces within a different range of controlled culture conditions. The physical constraints of the device in combination with perfusion enabled a more uniform aggregation of cells and microspheres, thus offering a potential strategy to control the clustering effect observed in static culture. Future experiments will be aimed at further exploring optimal conditions for modular tissue engineering which could be potentially translated to a scaled-up system for the manufacturing of larger quantity of bone tissue ex vivo.
